# Detection and diversity of viruses infecting African yam (*Dioscorea rotundata*) in a collection and F_1_ progenies in Côte d'Ivoire shed light to plant‐to‐plant viral transmission

**DOI:** 10.1111/ppa.13393

**Published:** 2021-05-14

**Authors:** Yacouba Bakayoko, Amani M. Kouakou, Abou B. Kouassi, Rose‐Marie Gomez, Konan E. B. Dibi, Brice S. Essis, Boni N’Zué, Patrick Adebola, Assanvon S.‐P. N’Guetta, Marie Umber

**Affiliations:** ^1^ Laboratoire de Biotechnologie Agriculture et Valorisation des Ressources Biologiques UFR Biosciences Université Félix Houphouët Boigny Abidjan Côte d'Ivoire; ^2^ Station de Recherche sur les Cultures Vivrières (SRCV Centre National de Recherche Agronomique Bouaké Côte d'Ivoire; ^3^ Unité de Recherche Agrosystèmes Tropicaux Institut National de Recherche pour l’Agriculture, l’Alimentation et l’Environnement Petit‐Bourg Guadeloupe France; ^4^ International Institut of Tropical Agriculture IITA‐Abuja Station Abuja Nigeria

**Keywords:** diagnosis, *Dioscorea rotundata*, molecular diversity, prevalence, yam breeding, yam viruses

## Abstract

Yam (*Dioscorea* spp.) is a major staple food whose production is hampered by viral diseases. However, the prevalence, diversity, transmission, and impact of yam‐infecting viruses remain poorly documented. This study reports on the symptomatology, prevalence, and molecular diversity of eight viruses in 38 *D*. *rotundata* accessions from a germplasm collection and 206 F_1_ hybrid progenies maintained in Côte d'Ivoire. Mean severity scores as assessed from leaf symptoms ranged from 2 to 4 in the germplasm collection and from 1 to 3 in F_1_ hybrids, respectively. Dioscorea mosaic‐associated virus (DMaV), potexviruses, and yam mosaic virus (YMV) were detected by PCR‐based diagnosis tools in single and mixed infections in both the *D*. *rotundata* collection and F_1_ progenies, whereas badnaviruses were detected only in the germplasm collection. In contrast, cucumber mosaic virus (CMV), yam macluraviruses, yam asymptomatic virus 1 (YaV1), and yam mild mosaic virus (YMMV) could not be detected. No correlation could be established between severity scores and indexing results. Phylogenetic analysis performed on partial viral sequences amplified from infected samples unveiled the presence of two putative novel viral species belonging to genera *Badnavirus* and *Potexvirus* and provided evidence for plant‐to‐plant transmission of YMV, DMaV, and yam potexviruses.

## Graphical abstract

A survey of leaf symptoms and viral molecular indexation on African yam germplasm and two hybrid populations unveiled a high prevalence of yam viruses in Côte d'Ivoire and provided evidence for their plant‐to‐plant transmission.

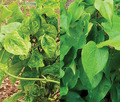

## 1 INTRODUCTION

Yam (*Dioscorea* spp.) is the second most important root and tuber crop after cassava and a major staple food in sub‐Saharan Africa, where it plays a vital role in food security for more than 60 million people. Five West African countries (Benin, Côte d'Ivoire [Ivory Coast], Ghana,Nigeria, and Togo) account for more than 95% of the worldwide yam production (FAOSTAT, 2020). In Côte d'Ivoire, yam is the leading staple crop with more than 7.2 million tonnes produced in 2018, with *D*. *rotundata* representing 75% of yam trade in the country (Touré et al., 2003). However, the production is insufficient, especially because yam yields have decreased from 8 to 5.5 t/ha in Côte d'Ivoire since 2007 (FAOSTAT, 2020) despite an increase in cultivated surface. This decrease is likely to result from the impact of viral diseases, the use of infected and/or too old planting material, soil fertility, and postharvest losses (Bakayoko et al., 2017).

Yams are primarily propagated vegetatively, leading to an accumulation of viruses resulting in multiple infections (Eni et al., 2008). Although the impact of viral diseases on yam production is poorly documented, decreases in yield and reduction of the quality of harvested tubers due to viral infections have been reported by Toualy et al. (2014), sometimes threatening entire productions. While infected tubers ensure the spread of viruses, several yam viruses are known to be transmitted by insects. Aphids are the vectors of yam mosaic virus (YMV; genus *Potyvirus*) and cucumber mosaic virus (CMV; genus *Cucumovirus*), while mealybugs are reported to transmit Dioscorea bacilliform virus (DBV; genus *Badnavirus*) (Odu et al., 2004). Using mainly viral metagenomics, a large number of novel viruses have been identified in yam during the last 15 years. Thus far, viruses belonging to genera *Ampelovirus*, *Aureusvirus*, *Badnavirus*, *Carlavirus*, *Cucumovirus*, *Fabavirus*, *Macluravirus*, *Potexvirus*, *Potyvirus*, *Sadwavirus*, and an unknown genus belonging to the family *Betaflexiviridae*, have been reported in yams (Umber et al., 2020).

In West Africa, five distinct viruses are usually detected in yam, including YMV, yam mild mosaic virus (YMMV; genus *Potyvirus*), CMV, DBV, and Dioscorea latent virus (DLV; genus *Potexvirus*) (Eni et al., 2008; Toualy et al., 2014). In Côte d'Ivoire, yam growers report increasing yield losses and observe virus‐like symptoms such as chlorosis, mosaic, deformation on leaves, and dwarfism (Séka et al., 2009; Toualy et al., 2014). As a consequence, farmers increase cultivated areas in order to compensate for yield losses and maintain production levels, resulting in conflicts over land use and to negative impacts on the environment.

The control of viral diseases affecting crops relies primarily on the use of clean seeds and/or the breeding of resistant varieties either by conventional methods or by genetic engineering. Yam sanitation programmes have been successfully implemented, resulting in the production of clean seeds (Umber et al., 2020). Such programmes rely on plant regeneration from meristem culture in vitro, thermotherapy, or cryotherapy (Filloux & Girard, 2006). The National Center for Agricultural Research (CNRA) works on the sustainability of yam production in Côte d'Ivoire by the selection and propagation of improved yam varieties that meet the expectations of the yam sector. These new hybrids have to combine high yield, good tuber quality, and especially resistance to yam mosaic disease, considering the high susceptibility of *D. rotundata* to viruses. Viral resistance is therefore an important trait for yam selection. The viral status of *D*. *rotundata* F_1_ progenies obtained from hybridizations by CNRA in Bouaké and of a diverse panel of *D*. *rotundata* accessions has been assessed using the comprehensive diagnostic scheme designed by the Biological Resources Center for Tropical Plants (BRC‐TP) located in Guadeloupe (French West Indies) (Umber et al., 2020). Molecular tools used for diagnosis include reverse transcription (RT)‐PCR for the detection of RNA viruses and immunocapture (IC)‐PCR for that of badnaviruses, which avoids false positives resulting from the presence of endogenous badnavirus sequences (eDBVs) in the genome of some yam species, including *D*. *rotundata* (Umber et al., 2014). RNA viruses targeted in this study include CMV, Dioscorea mosaic‐associated virus (DMaV; genus *Sadwavirus*), yam asymptomatic virus 1 (YaV1; genus *Ampelovirus*), YMV, and YMMV with specific tests. Yam potexviruses and yam macluraviruses are detected using generic tests, which are able to target several yam‐infecting viral species (Umber et al., 2020).

This study reports on the evaluation of the symptomatology, prevalence, and diversity of these viruses in a field germplasm collection of *D*. *rotundata* accessions and in the *D*. *rotundata* F_1_ progenies maintained in Côte d'Ivoire. The results suggest that the presence of yam‐infecting viruses in *D*. *rotundata* is not correlated with observed leaf symptoms and that plant‐to‐plant transmission occurs for at least some of the viruses. This work also reports on the first molecular detection of DMaV and potexviruses in yams in Africa using RT‐PCR‐based tests.

## 2 MATERIALS AND METHODS

### 2.1 Plant material

*D*. *rotundata* leaf samples were collected in October 2018 at the Food Crops Research Station of CNRA, located in central Côte d'Ivoire (7°46′N, 5°06′W, 376 m a.s.l.). Thirty‐eight accessions were selected according to various leaf symptoms from the CNRA germplasm collection, which was planted in March 2018 with a spacing of 1 m between and within rows. Two hundred and six F_1_ hybrids and their genitors were collected from a nearby plot. They originated from progenies of two biparental crosses involving *D*. *rotundata* genitors, one female (Cnraigr09/00001) and two males (TDr95/18555 and TDr00/00380). The first hybrid population of 70 individuals originated from the Cnraigr09/00001 × TDr99/18555 cross while the second population of 136 individuals resulted from the Cnraigr09/00001 × TDr00/00380 cross. This plot was planted in June 2018, 4 m away from the *D*. *rotundata* collection, and was organized in three completely randomized Fisher blocks with one replicate of each hybrid and each genitor per block. The sampling was carried out on the plant with the most important symptoms among the three replicates. Details of the samples are provided in Tables S1 and S2.

### 2.2 Assessment of leaf symptoms

Leaf symptoms were observed 3, 4, and 6 months after the planting of *D*. *rotundata* accessions and F_1_ hybrids. Symptom severity was assessed according to a rating scale established by the International Institute for Tropical Agriculture (IITA, 1998; Table 1). For the two progenies and their genitors, the score retained corresponds to that of the plant presenting the most important symptoms among the three replicates.

**TABLE 1:** Rating scale of viral symptom severity (IITA, 1998)

Score	Associated severity of viral symptoms
1	Symptomless plants
2	Plants presenting moderate symptoms (1%–25% of the leaves)
3	Plants presenting severe symptoms (26%–50% of the leaves)
4	Plants presenting very severe symptoms (51%–75% of the leaves)
5	Severely attacked plants presenting >75% distorted or malformed leaves and/or with signs of dwarfism

### 2.3 Molecular detection of yam viruses

Total nucleic acids (TNAs) were extracted from leaf samples according to procedure 2 developed by Foissac et al. (2005) and used for the detection by RT‐PCR of CMV, DMaV, YaV1, YMV, YMMV, yam macluraviruses, and yam potexviruses as described by Umber et al. (2020). Details of the primers are provided in Table S3. Badnaviruses were detected by immunocapture‐multiplex‐PCR (IC‐M‐PCR), as described by Umber et al. (2017), using BEL antiserum (Ndowora et al., 1999) and atpB1/B2 primers to control genomic DNA contamination (Soltis et al., 1999). All tests were performed twice in order to confirm the results.

### 2.4 Correlation between symptom severity and viral detection

Severity scores observed for *D*. *rotundata* accessions of the CNRA collection and the two F_1_ hybrid progenies were compared to indexing results. The corrplot package of the open source software R was used to calculate Pearson correlation coefficients between severity scores of viral symptoms and the results of viral indexing (R Core Team, 2021).

### 2.5 Analyses of the molecular diversity of yam viruses

PCR products amplified from *D*. *rotundata* accessions of the CNRA germplasm collection infected by potexviruses, badnaviruses, and/or DMaV were cloned into the pGEM‐T Easy vector (Promega) according to the manufacturer's instructions and sequenced (Genewiz). Nonredundant sequences were used for multiple alignments using the ClustalW component of MEGA X (Kumar et al., 2018). Phylogenetic trees were constructed using the maximum‐likelihood method and the robustness of the trees was determined using the bootstrap method with 1,000 replicates.

## 3 RESULTS

### 3.1 Severity scores based on leaf symptoms observed under field conditions

**FIGURE 1:**
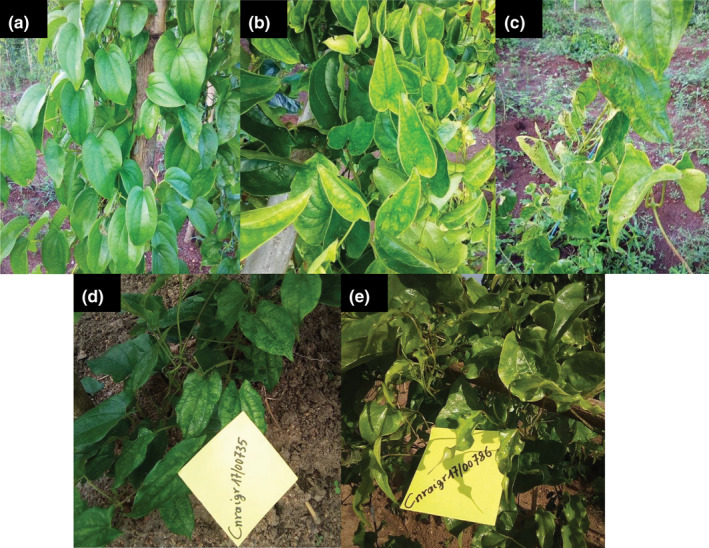
Viral symptoms observed on leaves of *Dioscorea* *rotundata* accessions from the CNRA collection and F_1_ hybrid populations. (a) Symptomless leaves of the accession CivcDr076. (b) Chlorotic symptoms observed on leaves of the accession CivcDr404. (c) Mosaic and puckering symptoms observed on leaves of the accession CicvDr409, associated with dwarfism. (d) Mosaic symptoms observed on leaves of the F_1_ hybrid Cnraigr17/00735. (e) Distortion symptoms observed on leaves of the F_1_ hybrid Cnraigr17/00786 [Colour figure can be viewed at wileyonlinelibrary.com]

Leaf symptoms including mosaic, dwarfism, leaf deformation, chlorosis, and puckering were observed on 37 of the 38 *D*. *rotundata* accessions used in this work, representing the variability of leaf symptoms displayed in the *D*. *rotundata* CNRA germplasm (Figure 1). Severity scores for symptoms ranged from 1 to 4, with one accession (2.4%), 15 (39.5%), 19 (50.0%), and 3 (7.9%), respectively (Table S1).

Six months after planting, leaf symptoms including mosaic, leaf curling, leaf deformations, and chlorosis were observed on the F_1_ hybrid progenies (Figure 1). Symptom severity scores ranged from 1 to 3 (Table S2). Most F_1_ hybrids (183/206; 88.8%) displayed moderate symptoms corresponding to severity score 2 whereas 18 hybrids (8.7%) scored 3, and only five (2.4%) were symptomless. In the progeny of the cross Cnraigr09/00001 × TDr95/18555, one of the 70 hybrids was symptomless (1.4%) whereas 66 displayed a severity score of 2 (94.3%) and three a severity score of 3 (4.3%). Within the progeny of the cross Cnraigr09/00001 × TDr00/00380, four hybrids out of 136 were symptomless (2.9%) whereas 117 displayed a severity score of 2 (86.0%), and 15 a severity score of 3 (11.0%). The female genitor Cnraigr09/00001 and male genitor TDr00/00380 displayed a severity score of 3, whereas the male genitor TDr95/18555 displaying a severity score of 2.

### 3.2 Virus prevalence in *D. rotundata* accessions and *D. rotundata* F_1_ progenies

**FIGURE 2:**
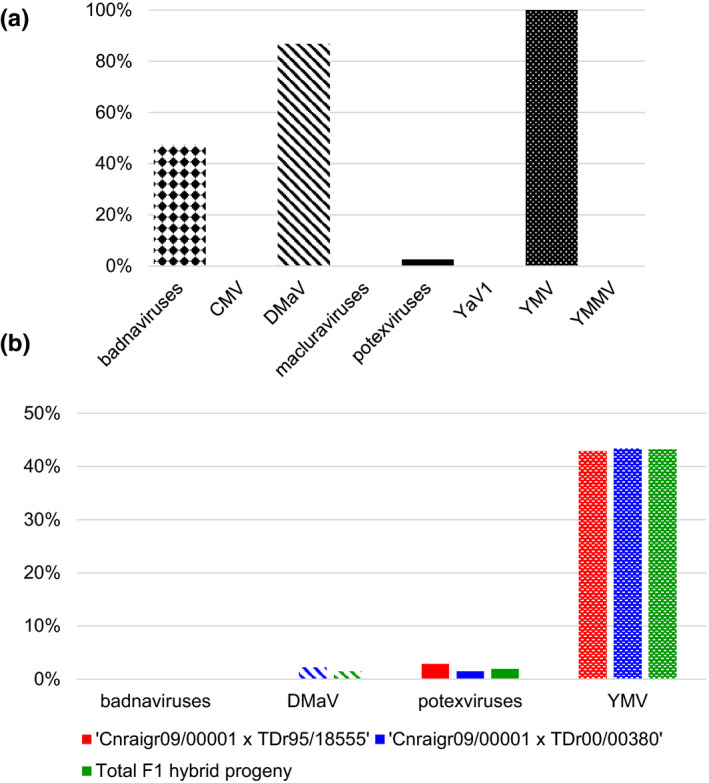
Prevalence of targeted yam viruses in *Dioscorea* *rotundata* samples. (a) Prevalence of eight viruses in the 38 accessions of the *D*. *rotundata* collection of CNRA. (b) Prevalence in each F_1_ progeny and in the total population of F_1_ hybrids. CMV: cucumber mosaic virus; DMaV: Dioscorea mosaic‐associated virus; YaV1: yam asymptomatic virus 1; YMV: yam mosaic virus; YMMV: yam mild mosaic virus [Colour figure can be viewed at wileyonlinelibrary.com]

YMV was detected in all 38 tested accessions, whereas 18 accessions (47.4%) were infected by badnaviruses, 33 (86.8%) by DMaV, and one (2.6%) by potexviruses (Figure 2a). CMV, YaV1, YMMV, and yam macluraviruses were not detected in the tested accessions. Coinfections by two or more distinct viruses occurred in all tested accessions: 22/38 (57.9%) were infected by two viruses and 15/38 (39.5%) by three viruses. Most of the coinfections (33/38, 86.8%) involved YMV and DMaV (Table 2).

**TABLE 2:** Numbers of plants and percentages of coinfections by different viruses in the *Dioscorea rotundata* germplasm and the F_1_ hybrid progenies

Type of coinfection	Targeted viruses	*D*. *rotundata* collection	F_1_ hybrid progenies
Double Infection	YMV + DMaV	18/38 (47.4%)	2/206 (1.0%)
	YMV + potexvirus	0/38 (0.0%)	1/206 (0.5%)
	YMV + badnavirus	4/38 (10.5%)	0/206 (0.0%)
Triple infection	YMV + DMaV + badnavirus	14/38 (36.8%)	0/206 (0.0%)
	YMV + DMaV + potexvirus	1/38 (2.6%)	0/206 (0.0%)

Diagnosis was carried out on 206 *D*. *rotundata* F_1_ hybrid progenies for the four viruses detected in *D*. *rotundata* accessions of the CNRA germplasm collection (badnaviruses, DMaV, potexviruses, and YMV), assuming that the collection was the source of viral transmission. Ninety‐nine F_1_ hybrids (48.1%) were infected by at least one virus, including 32/70 (45.7%) and 67/136 (49.26%) hybrids from the crosses Cnraigr09/00001 × TDr99/18555 and Cnraigr09/00001 × TDr00/00380, respectively (Table S2). Only YMV and potexviruses were detected in the progeny Cnraigr09/00001 × TDr99/18555 with prevalences of 42.9% and 2.9%, respectively (Figure 2b). Hybrids from the cross Cnraigr09/00001 × TDr00/00380 were infected by YMV (43.4%) and potexviruses (1.5%), but also by DMaV (2.2%). Badnaviruses were not detected in progenies of either cross. Coinfections were found in three hybrids (1.5%) and involved all three detected viruses (Table 2).

### 3.3 Correlation between severity scores of leaf symptoms and detected viruses

**FIGURE 3:**
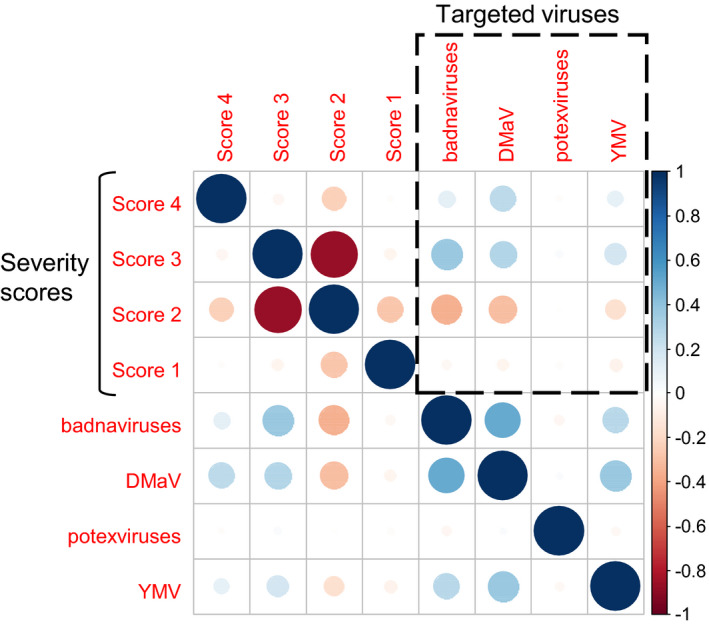
Correlogram obtained using Pearson's matrix correlation coefficients between severity scores based on leaf symptoms and detected viruses. Blue and red spots represent positive and negative correlations, respectively. Dotted black boxed section displays the results of possible correlations between severity scores and the type of detected viruses [Colour figure can be viewed at wileyonlinelibrary.com]

No overall correlation could be established between symptom severity scores and the detection of a particular virus (Figure 3). For instance, YMV was detected in the F_1_ hybrids Cnraigr17/00096 and Cnraigr17/00669, which were symptomless. In contrast, no virus was detected in 99 out of the 183 F_1_ hybrids (54.1%) displaying a score of 2, nor in 11 out of the 18 (61.1%) of those displaying a score of 3 (11/18).

Overall, the correlogram revealed moderate correlations between viral infections and severity scores, regardless of the virus considered (Table 3). Indeed, the detection of DMaV was positively correlated with severity scores 3 and 4 (*r* = 0.3; *p* < 0.0001) and negatively correlated with score 2 (*r* = −0.309; *p* < 0.0001), with high significance. Similarly, the presence of badnaviruses was positively associated with severity score 3 (*r* = 0.377; *p* < 0.0001) and negatively correlated with severity score 2 (*r* = −0.351; *p* < 0.0001), with high significance.

**TABLE 3:** Pearson's correlation coefficients between severity scores of leaf symptoms and detected viruses

Severity score	Detected viruses			
	Badnavirus	DMaV	Potexvirus	YMV
4	0.111	0.268***	−0.016	0.107
3	0.377***	0.291***	0.023	0.182*
2	−0.351***	−0.309***	−0.006	−0.160*
1	−0.036	−0.054	−0.019	−0.070

Pearson's correlation coefficients are different from 0 at significance level α = .05; ***highly significant; *significant.

### 3.4 Molecular diversity of badnaviruses, DMaV, and potexviruses identified in Bouaké

**FIGURE 4:**
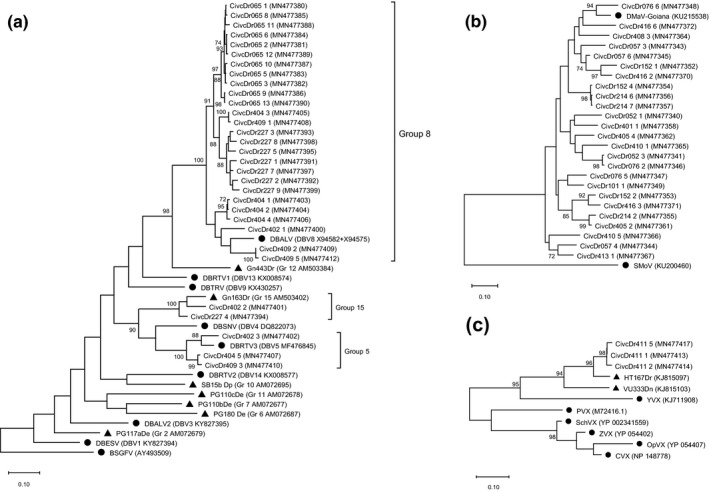
Maximum‐likelihood phylogenetic trees of partial sequences of viruses detected in *Dioscorea* *rotundata* accessions of the CNRA collection. Bootstrap values from 1,000 replicates are shown at nodes when above 70%. GenBank accession numbers are provided. (a) Phylogenetic tree of badnaviruses based on RT‐RNase H domain: black circles represent the corresponding domain of the eight complete yam badnavirus genomes and the genome of banana streak GF virus (BSGFV), used as an outgroup; black triangles correspond to yam badnavirus partial sequences available from GenBank. Yam badnavirus groups are shown. (b) Phylogenetic tree of Dioscorea mosaic‐associated virus (DMaV) based on RdRp domain: the black circles correspond to the studied part of the RNA1 of the genomes of DMaV from Brazil and of strawberry mottle virus (SMoV) used as an outgroup. (c) Phylogenetic tree of potexviruses based on RdRp domain: black circles represent the corresponding domain of the yam virus X (YVX) genome and the genomes of five potexviruses used for the analysis (PVX: potato virus X, SchVX: Schlumbergera virus X, ZVX: Zygocactus virus X, OpVX: Opuntia virus X, and CVX: cactus virus X); the black triangles correspond to yam potexvirus partial sequences available from GenBank

Thirty‐one nonredundant nucleotide sequences of badnaviruses were amplified from five *D*. *rotundata* accessions (accession numbers MN477380–MN477412). Phylogenetic analyses showed that they belong to Groups 5, 8, and 15 defined by Kenyon et al. (2008) and Bömer et al. (2016) (Figure 4a). Twenty‐five nonredundant nucleotide sequences were obtained from 12 DMaV‐infected accessions (accession numbers MN477340–MN477372). Phylogenetic analyses showed that they are homologous to the sequence reported by Hayashi et al. (2017), sharing between 78.2% and 87.6% of identity with DMaV‐Goiana (Figure 4b). Seven nonredundant nucleotide sequences were generated from the only potexvirus‐infected accession CivcDr411 (accession numbers MN477413–MN477423). They were expressed in three nonredundant protein sequences as described by Acina‐Mambole et al. (2014) and formed a distinct group from the three potexvirus species previously identified in yam (Figure 4c).

## 4 DISCUSSION

The vast majority of the *D*. *rotundata* accessions of the CNRA collection expressed leaf symptoms associated with viral diseases, the most common ones being puckering, chlorosis, and mosaic. The average severity score based on leaf symptoms was moderate but some accessions presented very high scores, reaching 4. Toualy et al. (2014) reported similar severity scores ranging from 3 to 4 for virus‐like symptoms in 73% of yam plants evaluated across Côte d'Ivoire. These high scores are probably due to the ever‐increasing viral load in the planting material of the *D*. *rotundata* accessions in Bouaké during the course of several decades. The Togolese Institute of Agronomic Research (ITRA) reported that, in the collection of *D*. *cayenensis*‐*rotundata* accessions in Togo, local landraces are highly susceptible to viral diseases (ITRA, 2003). The presence of various virus‐like symptoms on the leaves of *D*. *rotundata* accessions could reveal the sensitivity of this yam species to viruses and the efficiency of viral transmission, considering how early symptoms were observed on F_1_ hybrid progenies.

Four different viruses, including badnaviruses, DMaV, potexviruses, and YMV, were detected in *D*. *rotundata* accessions of the CNRA germplasm collection. YMV and badnaviruses were reported for many years in *D*. *rotundata* cultivars in Africa including Côte d'Ivoire (Eni et al., 2008). Thus, Adjata (1991) detected YMV in 87% of local *D*. *rotundata* cultivars in Togo using ELISA tests. In the present study, YMV was detected in all 38 studied *D*. *rotundata* accessions and badnaviruses in 47% of them. This result differs from that of Toualy et al. (2014) who indicated that badnaviruses were more prevalent than YMV in 486 tested yam accessions. This could be explained by the molecular tools used in this study, especially for badnaviruses, for which the presence of endogenous viral sequences in the yam genome was not considered. However, the current prevalence of YMV (100%) is close to that (88%) obtained by Séka et al. (2009) in the Bouaké area.

Potexviruses and DMaV are present in the analysed *D*. *rotundata* accessions. Potexviruses were previously reported in Africa using serological tests, but not in *D*. *rotundata* (Phillips et al., 1986). Likewise, this is the first survey on DMaV presence in Africa, although DMaV sequences had been obtained by high‐throughput sequencing in one yam plant from Nigeria (Silva et al., 2019). In contrast, CMV was not detected in any of the 38 tested *D*. *rotundata* accessions. Séka et al. (2009) reported a low prevalence (1.5%) of CMV in the Bouaké area, whereas Thottappilly (1992) indicated that CMV only infects *D*. *alata* in Côte d'Ivoire, which could explain the absence of this virus in the analysed *D*. *rotundata* samples. YMMV and YaV1 were also not detected in the analysed *D*. *rotundata* samples. However, YMMV was previously reported in *D*. *cayenensis*‐*rotundata* in Africa with a low prevalence (Bousalem et al., 2003), whereas YaV1 is probably present in Africa because two expressed sequence tag (EST) sequences from a *D*. *alata* plant from Nigeria displayed 85.5% and 90.4% of homology to the YaV1 genome sequence (Marais et al., 2020). No macluravirus was detected, confirming results from Lan et al. (2018) who claimed that none of the species of macluravirus identified in yam have yet been reported in Africa.

Severity scores based on leaf symptoms ranged from 1 to 3 in the F_1_ hybrid progenies. According to the classification determined by IITA (1998), a yam genotype is considered resistant to viruses when the severity score is less than or equal to 2 and a genotype presenting a symptom severity score greater than 2 is considered susceptible. Thus, about 91.3% of the hybrid progenies from this study would be considered as resistant, and these results are similar to those obtained by Mignouna et al. (2001) in Benin for a F_1_ hybrid population from two local accessions of *D*. *rotundata*. However, they are inconsistent with molecular detection results that revealed the presence of viruses in 41.7% of the F_1_ hybrids with low severity scores, indicating that severity scores observed in 1‐year‐old F_1_ hybrid progenies are not a reliable method to evaluate viral resistance in a breeding programme.

As infected plants could be symptomless and some virus‐free plants could display virus‐like symptoms due to mineral deficiency, for example, appropriate tools have been implemented to detect viruses in order to ensure the diagnosis of viral infection. For this purpose, PCR‐based diagnostic tools are more sensitive than serological tests but their effectiveness depends on the good quality of nucleic acids, especially for RNA templates. The procedure 2 for extraction of total nucleic acids, proposed by Foissac et al. (2005), preserves the integrity of RNA and ensures the sensitivity of the RT‐PCR. Thus, among the five symptomless F_1_ hybrids (severity score = 1), the genotypes Cnraigr17/00096 and Cnraigr17/00669 were infected by YMV. In similar studies, YMV was detected in symptomless F_1_ hybrids, and these genotypes have been considered tolerant (Mignouna et al., 2001). Tolerance, or partial resistance, could be the basis for resistance in field conditions and protection of plants over long periods (Lecoq et al., 2004). Several *D*. *rotundata* accessions and F_1_ hybrids were coinfected by two or more viruses. Rates of double and triple infections were significantly higher in *D*. *rotundata* accessions than in F_1_ hybrid progenies. This may be the result of the accumulation of viruses over time and consecutive between‐plant transmission events in the CNRA collection, which has been established since 1968, while the F_1_ offspring were only transferred to the field for one year at the time of sampling. Finally, 11 of the F_1_ hybrids displaying the high severity score 3 were not infected by any of the targeted viruses. Virus‐like symptoms on yam leaves could be caused by nutrition deficiencies, or by other yam viruses that were not targeted by the detection tools used in the present study. Indeed, since the field trials were conducted and plants indexed, new yam viruses have been characterized, like yam virus Y (YVY), belonging to the *Betaflexiviridae* family, which seems to be prevalent in Africa (Silva et al., 2019). In addition, primers used for detection tests could fail to target some divergent isolates or tests could be not sensitive enough to detect low viral titre, and explain the negative results.

In order to develop a new preventive control strategy, adapted to existing conditions, data on the relationship between the severity of viral infections and the type of detected viruses is essential. Thus, statistical analyses showed positive but moderate correlations between severity scores 3 and 4 and the presence of badnaviruses and DMaV, meaning that plant infection by these two viruses leads to severe leaf symptoms, although this result could be due to coinfections, regarding the high prevalence of DMaV and YMV. As expected, their detection was negatively and significantly correlated with severity scores 2 and 1. In contrast, the correlation between YMV and symptom severity scores was very weak, because this virus infected all the accessions of the CNRA collection, as well as more than 40% of the F_1_ hybrid progenies. Thus, the highest severity scores (3 and 4) could rather result from a synergy of the coinfections by YMV and badnaviruses or DMaV. However, even though significant, correlation rates were very weak. As Njukeng et al. (2014) argued, prediction of the presence of a type of virus based on severity scores is therefore impossible on yam crop.

As the genetic diversity of YMV, which is the most widespread virus in yam crop in West Africa, has already been assessed worldwide (Bousalem et al., 2000), only that of DMaV, badnaviruses, and potexviruses sequences was analysed. The molecular analyses of these viruses detected from the CNRA *D*. *rotundata* collection showed a different structure of diversity depending on targeted viruses, and revealed new viral species.

Regarding the badnaviruses, their sequences belong to three different phylogenetic groups representing three distinct species. Most of these sequences (26 out 31) fit to Group 8, which is the most widespread species of Dioscorea bacilliform virus (DBV) worldwide (Kenyon et al., 2008). Dioscorea bacilliform AL virus (DBALV), belonging to the DBV8 species, was the first badnavirus genome to be fully characterized from yam (Briddon et al., 1999). Whilst it was first detected in *D*. *alata*, it is now known to infect other yam species, such as *D*. *bulbifera*, *D*. *nummularia*, *D*. *rotundata*, and *D*. *trifida* (Kenyon et al., 2008; Sukal et al., 2020; Umber et al., 2017). Three of the badnaviral sequences obtained in this study clustered with Group 5 (DBV5), of which the complete genome of the corresponding species, named Dioscorea bacilliform RT virus 3 (DBRTV3), has been recently sequenced in Nigeria from *D*. *rotundata* (Bömer et al., 2018). Endogenous viral sequences from DBV8 and DBV5 species are known to be integrated into the genome of yam species of the *D*. *cayenensis*‐*rotundata* complex (Umber et al., 2014). Some viral insertions are supposed to lead to viral resistance through posttranscriptional gene silencing, which prevents further plant infection with corresponding viral genomes (Chabannes et al., 2013). However, detection of DBALV and DBRTV3 in episomal form in *D*. *rotundata* shows the opposite, and confirms that these endogenous sequences are not involved in viral resistance in this yam species (Umber et al., 2017). The two remaining sequences, CivcDr227_4 and CivcDr402_2, share 88% to 93% of identity with six partial badnaviral sequences identified in Africa belonging to Group 15 (DBV15), from *D*. *rotundata* and *D*. *alata* (Bömer et al., 2016; Eni et al., 2008). However, these two sequences share 60% to 78% of identity with RT‐RNase H domains of the eight available complete genomes of yam badnaviruses. Because 80% of identity in RT‐RNAse H domain is the threshold for the demarcation of species within the *Badnavirus* genus (Teycheney et al., 2020), these sequences belong to a yam badnavirus species for which no complete genome has yet been sequenced. Further studies must be implemented in order to characterize this species, which seems to specifically infect yam crops in Africa. Finally, mixed infections by members of different badnavirus species were observed, as previously reported by Umber et al. (2017), because the CivcDr227 accession hosted sequences belonging to DBV8 and DBV15, and CivcDr404 and CivcDr409 to DBV8 and DBV5, whereas CivcDr402 was infected by all three above‐mentioned species.

DMaV has been recently characterized in Brazil by metagenomics (Hayashi et al., 2017) and belongs to the genus *Sadwavirus* (Sanfaçon et al., 2020). DMaV isolates identified in this study form a single species with isolates originating from the American continent; thus, we provide evidence here that this viral species is present in both continents.

The potexvirus strain detected in the single potexvirus‐infected accession of *D*. *rotundata* forms a distinct phylogenetic group from the three known species identified in yam, including the HT167Dr sequence associated to the yam potexvirus 1 group. Indeed, the potexviral sequences generated in this study share 89.9% to 91.3% of identity with the HT167Dr protein sequence. This level of homology is in the same range as that between the corresponding domain of cactus virus X, Opuntia virus X, Schlumbergera virus X, and Zygocactus virus X (84.1% to 92.8%), which are considered as distinct species. Thus, the potexvirus strain detected in the CNRA collection of *D*. *rotundata* is probably a new species. Further analyses are needed to confirm this assumption, especially because of the small size of the fragment considered. Nevertheless, the identification of this putative new species shows that the molecular tool developed by the BRC‐TP in Guadeloupe is able to detect a large diversity of potexviruses infecting yam (Umber et al., 2020).

Four types of viruses, namely YMV, DMaV, badnaviruses, and potexviruses, were detected in 100%, 86.8%, 47.4%, and 2.6% of the 38 tested accessions of *D*. *rotundata*, respectively, and are likely to be the local source of viral inoculum for the F_1_ progenies. However, only three viruses were detected in the two F_1_ hybrid populations, with prevalences of 42.9% and 43.4% for YMV, 0% and 2.2% for DMaV, and 2.9% and 1.5% for potexviruses, respectively. Interestingly, YMV and DMaV, which are both highly prevalent in the collection, were detected in F_1_ hybrids in very different proportions. Based on viral prevalence in *D*. *rotundata* accessions and the F_1_ progenies, a relative transmission rate could be calculated, without considering the putative viral resistance of certain genotypes. Thus, after 3 months of planting, badnaviruses are not transmitted or not detected because the test for badnaviruses detection by IC‐PCR could fail to detect low viral titre, and estimates of the transmission rates are 1.7% for DMaV, 43.2% for YMV, and 73.1% for potexviruses. These different transmission rates could be related to the type of vector that transmits these different viruses. Indeed, aphids, which are the vector in natural transmission of the genus *Potyvirus*, are flying insects, whereas badnaviruses are transmitted by mealybugs (Odu et al., 2004), which need to be transported from plant to plant by ants. DMaV belongs to the family *Secovirideae*, whose members are transmitted by insects or soilborne nematodes (Thompson et al., 2017). For instance, grapevine fanleaf virus (GFLV), a nepovirus, is transmitted by the nematode *Xiphinema index* (Schellenberger et al., 2011). Nematode‐mediated viral transmission could result in high infection in the field if the same crop is planted in the same infested plot over the years. In contrast, crop rotation leads to natural sanitation of the infected soil (Bilevai et al., 2009). The plot supporting the F_1_ hybrid progenies was previously covered by a pepper crop and that may explain the weak DMaV contamination of that population, assuming pepper plants would not be the host for the yam‐feeding nematodes. Finally, while seed transmission has been reported for potyviruses (Johansen et al., 1994), this mode of transmission has never been demonstrated for YMV. However, because the female parental accession Cnraigr09/00001 was infected by YMV (Table S2), seed transmission of this virus could explain the high infection rate to its 1‐year‐old progeny.

This study evaluated the occurrence and diversity of several viruses in the CNRA in vivo collection of *D*. *rotundata*, conserved for the long‐term at the Food Crop Research Station in Bouaké, and in two 1‐year‐old F_1_ hybrid populations. Four virus taxa were detected within the yam collection, including potexviruses and DMaV, which have never been reported in Côte d'Ivoire before. YMV, DMaV, and badnaviruses had high prevalences and frequently occurred in mixed infections. These results show the necessity of implementing a sanitation programme for the CNRA yam collection. Furthermore, there were highly significant but weak correlations between severity scores of viral symptoms and PCR‐based detection of YMV and badnaviruses. Thus, for an accurate evaluation of viral diseases in yam crops, severity scores based on viral symptoms must be combined with effective PCR‐based diagnosis tests. Finally, phylogenetic analyses highlighted two new putative viral species, belonging to the genera *Badnavirus* and *Potexvirus*. This study highlights the great diversity of viruses infecting yams worldwide and the challenge breeders and virologists encounter in the selection of resistant yam varieties. It also gives a basis for investigations in order to understand the transmission pattern of viral diseases in yam.

## CONFLICTS OF INTEREST

The authors declare no conflict of interest.

## AUTHOR CONTRIBUTIONS

Y.B., A.M.K., B.N., P.A., A.S.‐P.N., and M.U. conceived the experiments; Y.B., B.S.E., R.‐M.G., and M.U. performed the experiments; Y.B., K.E.B.D., and M.U. analysed the data; Y.B., A.M.K., A.B.K., and M.U. wrote the manuscript.

## Supporting information

Table S1

Table S2

Table S3

## Data Availability

The nucleotide sequences reported in this work have been deposited in the GenBank database under accession numbers MN477340–MN477372 and MN477380–MN477423.

## References

[ppa13393-bib-0022] Acina‐Mambole, I., Bonheur, L., Svanella‐Dumas, L., Filloux, D., Gomez, R.‐M., Faure, C. et al (2014) Molecular characterization of yam virus X, a new potexvirus infecting yams (*Dioscorea* spp) and evidence for the existence of at least three distinct potexviruses infecting yams. Archives of Virology, 159, 3421–3426. 25204410 10.1007/s00705-014-2211-3

[ppa13393-bib-0001] Adjata, K.D. (1991) Application du test Immuno‐enzymatique (test ELISA) à la détection des potyvirus de l’igname (*Dioscorea* spp). [Application of enzyme‐linked immunosorbent assay (ELISA) to the detection of yam potyviruses (*Dioscorea* spp)]. End of agronomic studies thesis, no. 90/01/PV. Lomé, Togo: E.S.A.‐ UL.

[ppa13393-bib-0002] Bakayoko, G.A., Kouamé, K.F. & Boraud, N.K.M. (2017) Culture de l’igname au Centre‐Est de la Côte d’Ivoire: contraintes, caractéristiques sociodémographiques et agronomiques. Journal of Applied Biosciences, 110, 10701–10713.

[ppa13393-bib-0003] Bilevai, T., Choleva, B., Hockland, S. & Ciancio, A. (2009) Management of virus‐transmitting nematodes with special emphasis on south‐east Europe. In: Ciancio, A. & Mukerji, K. (Eds.) Integrated management of fruit crops and forest nematodes. *Integrated management of plant pests and diseases* . 4, Dordrecht, Netherlands: Springer, pp. 215–242.

[ppa13393-bib-0004] Bömer, M., Rathnayake, A.I., Visendi, P., Silva, G. & Seal, S.E. (2018) Complete genome sequence of a new member of the genus *Badnavirus*, Dioscorea bacilliform RT virus 3, reveals the first evidence of recombination in yam badnaviruses. Archives of Virology, 163, 533–538. 29134336 10.1007/s00705-017-3605-9 PMC5799344

[ppa13393-bib-0005] Bömer, M., Turaki, A.A., Silva, G., Kumar, P.L. & Seal, S.E. (2016) A sequence‐independent strategy for amplification and characterisation of episomal badnavirus sequences reveals three previously uncharacterised yam badnaviruses. Viruses, 8, 188. 10.3390/v8070188 PMC4974523 27399761

[ppa13393-bib-0006] Bousalem, M., Dallot, S., Fuji, S. & Natsuaki, K.T. (2003) Origin, world‐wide dispersion, bio‐geographical diversification, radiation and recombination: An evolutionary history of Yam mild mosaic virus (YMMV). Infection, Genetics and Evolution, 3, 189–206. 10.1016/s1567-1348(03)00085-6 14522183

[ppa13393-bib-0007] Bousalem, M., Douzery, E.J.P. & Fargette, D. (2000) High genetic diversity, distant phylogenetic relationships and intraspecies recombination events among natural populations of yam mosaic virus: a contribution to understanding potyvirus evolution. Journal of General Virology, 81, 243–255. 10.1099/0022-1317-81-1-243 10640564

[ppa13393-bib-0008] Briddon, R.W., Phillips, S., Brunt, A. & Hull, R. (1999) Analysis of the sequence of *Dioscorea alata bacilliform virus*; comparison to other members of the badnavirus group. Virus Genes, 18, 277–283. 10456795 10.1023/a:1008076420783

[ppa13393-bib-0009] Chabannes, M., Duroy, P.‐O., Seguin, J., Rajendran, R., Laboureau, N., Pooggin, M. et al (2013) Banana plants use post‐transcriptional gene silencing to control banana streak virus infection. In: *14èmes Rencontres de Virologie Végétale*, Aussois, France, 13–17 January 2013. Paris, France: INRA, p. 87.

[ppa13393-bib-0010] Eni, A.O. & Hughes, J.d’A. & Rey, M.E.C., (2008) Survey of incidence and distribution of five viruses infecting yams in major yam‐producing zones in Benin. Journal of Applied Biosciences, 153, 223–232.

[ppa13393-bib-0011] FAOSTAT (2020) Food and agriculture data. Available at: http://www.fao.org/faostat/ [Accessed 13 May 2020].

[ppa13393-bib-0012] Filloux, D. & Girard, J.‐C. (2006) Indexing and elimination of viruses infecting yams (*Dioscorea* spp.) for the safe movement of germplasm. In: *14th Triennial Symposium of the ISTRC*. Triruvananthapuram, Kerala, India: International Society for Tropical Root Crops, p. 13.

[ppa13393-bib-0013] Foissac, X., Svanella‐Dumas, L., Gentit, P., Dulucq, M.J., Marais, A. & Candresse, T. (2005) Polyvalent degenerate oligonucleotides reverse transcription‐polymerase chain reaction: a polyvalent detection and characterization tool for trichoviruses, capilloviruses, and foveaviruses. Phytopathology, 95, 617–625. 18943777 10.1094/PHYTO-95-0617

[ppa13393-bib-0014] Hayashi, E.A.I., Blawid, R., de Melo, F.L., Andrade, M.S., Pio‐Ribeiro, G., de Andrade, G.P. et al (2017) Complete genome sequence of a putative new secovirus infecting yam (*Dioscorea*) plants. Archives of Virology, 162, 317–319. 27730382 10.1007/s00705-016-3104-4

[ppa13393-bib-0015] IITA (1998) Annual report of project 13: Improvement of Yam base systems. Ibadan, Nigeria: International Institute of Tropical Agriculture.

[ppa13393-bib-0016] ITRA (2003) Rapport annuel 2003: Cultures vivrières. Lomé, Togo: Ministère de l’Agriculture, de l’Élevage et de la Pêche, p. 64.

[ppa13393-bib-0017] Johansen, E., Edwards, M.C. & Hampton, R.O. (1994) Seed transmission of viruses: Current perspectives. Annual Review of Phytopathology, 32, 363–386.

[ppa13393-bib-0018] Kenyon, L., Lebas, B.S.M. & Seal, S.E. (2008) Yams (*Dioscorea spp*.) from the South Pacific Islands contain many novel badnaviruses: Implications for international movement of yam germplasm. Archives of Virology, 153, 877–889. 18330495 10.1007/s00705-008-0062-5

[ppa13393-bib-0019] Kumar, S., Stecher, G., Li, M., Knyaz, C. & Tamura, K. (2018) MEGA X: Molecular evolutionary genetics analysis across computing platforms. Molecular Biology and Evolution, 35, 1547–1549. 29722887 10.1093/molbev/msy096 PMC5967553

[ppa13393-bib-0020] Lan, P., Meng, Y.u., Shen, P., Li, R., Ma, Y., Tan, S. et al (2018) Complete genome sequence of yam chlorotic necrosis virus, a novel macluravirus infecting yam. Archives of Virology, 163, 2275–2278. 29680924 10.1007/s00705-018-3851-5

[ppa13393-bib-0021] Lecoq, H., Moury, B., Desbiez, C., Palloix, A. & Pitrat, M. (2004) Durable virus resistance in plants through conventional approaches: A challenge. Virus Research, 100, 31–39. 15036833 10.1016/j.virusres.2003.12.012

[ppa13393-bib-0023] Marais, A., Umber, M., Filloux, D., Gomez, R.‐M., Faure, C., Pavis, C. et al (2020) Yam asymptomatic virus 1, a novel virus infecting yams (*Dioscorea* spp.) with significant prevalence in a germplasm collection. Archives of Virology, 165, 2653–2657. 32852617 10.1007/s00705-020-04787-0

[ppa13393-bib-0024] Mignouna, H.D., Njukeng, P., Abang, M.M. & Asiedu, R. (2001) Inheritance of resistance to *Yam mosaic virus*, genus *Potyvirus* in white yam (*Dioscorea rotundata*). Theoretical and Applied Genetics, 103, 1196–2000.

[ppa13393-bib-0025] Ndowora, T., Dahal, G., LaFleur, D., Harper, G., Hull, R., Olszewski, N.E. et al (1999) Evidence that badnavirus infection in *Musa* can originate from integrated pararetroviral sequences. Virology, 255, 214–220. 10069946 10.1006/viro.1998.9582

[ppa13393-bib-0026] Njukeng, A.P., Azeteh, I.N. & Mbong, G.A. (2014) Survey of the incidence and distribution of two virus infecting yam (*Dioscorea* spp) in two agro‐ecological zones of Cameroon. International Journal of Current Microbiology and Applied Sciences, 3, 1153–1166.

[ppa13393-bib-0027] Odu, B.O., Hughes, J.d'A., Asiedu, R., Ng, N.Q., Shoyinka, S.A. & Oladiran, O.A. (2004) Responses of white yam (*Dioscorea rotundata*) cultivars to inoculation with three viruses. Plant Pathology, 53, 141–147.

[ppa13393-bib-0028] Phillips, S., Piggott, J.d'a. & Brunt, A.A. (1986) Further evidence that dioscorea latent virus is a potexvirus. Annals of Applied Biology, 109, 137–145.

[ppa13393-bib-0029] R Core Team (2021). R: A Language and Environment for Statistical Computing. Vienna, Austria: R Foundation for Statistical Computing. Available at: https://www.R‐project.org/ [Accessed 21 April 2021].

[ppa13393-bib-0030] Sanfaçon, H., Dasgupta, I., Fuchs, M., Karasev, A.V., Petrzik, K., Thompson, J.R. et al (2020) Proposed revision of the family *Secoviridae* taxonomy to create three subgenera, “*Satsumavirus*”, “*Stramovirus*” and “*Cholivirus*”, in the genus *Sadwavirus* . Archives of Virology, 165, 527–533. 31848707 10.1007/s00705-019-04468-7

[ppa13393-bib-0031] Schellenberger, P., Sauter, C., Lorber, B., Bron, P., Trapani, S., Bergdoll, M. et al (2011) Structural insights into viral determinants of nematode mediated *Grapevine fanleaf virus* transmission. PLoS Pathogens, 7, e1002034. 21625570 10.1371/journal.ppat.1002034 PMC3098200

[ppa13393-bib-0032] Séka, K., Diallo, A.H., Kouassi, N.K. & Aké, S. (2009) Incidence du *Yam mosaic virus* (YMV) et du *Cucumber mosaic virus* (CMV) sur des variétés de *Dioscorea* spp. cultivées dans les régions de Bouaké et de Toumodi en Côte d'Ivoire. International Journal of Current Microbiology and Applied Sciences, 3, 694–703.

[ppa13393-bib-0033] Silva, G., Bömer, M., Rathnayake, A.I., Sewe, S.O., Visendi, P., Oyekanmi, J.O. et al (2019) Molecular characterization of a new virus species identified in yam (*Dioscorea* spp.) by high‐throughput sequencing. Plants, 8, 167. 10.3390/plants8060167 PMC6630666 31212654

[ppa13393-bib-0034] Soltis, P.S., Soltis, D.E. & Chase, M.W. (1999) Angiosperm phylogeny inferred from multiple genes as a tool for comparative biology. Nature, 402, 402–404. 10586878 10.1038/46528

[ppa13393-bib-0035] Sukal, A.C., Kidanemariam, D.B., Dale, J.L., Harding, R.M. & James, A.P. (2020) Characterization and genetic diversity of Dioscorea bacilliform viruses present in a Pacific yam germplasm collection. Plant Pathology, 69, 576–584.

[ppa13393-bib-0036] Teycheney, P.‐Y., Geering, A.D.W., Dasgupta, I., Hull, R., Kreuze, J.F., Lockhart, B. et al (2020) ICTV virus taxonomy profile: *Caulimoviridae* . Journal of General Virology, 101, 1025–1026. 10.1099/jgv.0.001497 PMC7660458 32940596

[ppa13393-bib-0037] Thompson, J.R., Dasgupta, I., Fuchs, M., Iwanami, T., Karasev, A.V., Petrzik, K. et al (2017) ICTV virus taxonomy profile: *Secoviridae* . Journal of General Virology, 98, 529. 10.1099/jgv.0.000779 PMC5657025 28452295

[ppa13393-bib-0038] Thottappilly, G. (1992) Plant virus diseases of importance to African agriculture. Journal of Phytopathology, 134, 265–288.

[ppa13393-bib-0039] Toualy, M.N.Y., Diallo, A.H., Akinbade, S.A., Séka, K. & Kumar, P.L.(2014) Distribution, incidence and severity of viral diseases of yam (*Dioscorea* spp.) in Côte d’Ivoire. African Journal of Biotechnology, 13, 465–470.

[ppa13393-bib-0040] Touré, M., Stessens, J., Zohouri, G.P. & Tollens, E. (2003) Sociologie des réseaux de commercialisation sur le marché de gros de Bouaké (Côte d’Ivoire): Le cas des grossistes d’igname. Cotonou, Bénin: Séminaire international post‐récolte et consommation des ignames.

[ppa13393-bib-0041] Umber, M., Filloux, D., Gélabale, S., Gomez, R.‐M., Marais, A., Gallet, S. et al (2020) Molecular viral diagnosis and sanitation of yam genetic resources: Implications for safe yam germplasm exchange. Viruses, 12, 1101. 10.3390/v12101101 PMC7650539 33003342

[ppa13393-bib-0042] Umber, M., Filloux, D., Muller, E., Laboureau, N., Galzi, S., Roumagnac, P. et al (2014) The genome of African yam (*Dioscorea cayenensis‐rotundata* complex) hosts endogenous sequences from four distinct badnavirus species. Molecular Plant Pathology, 15, 790–801. 24605894 10.1111/mpp.12137 PMC6638810

[ppa13393-bib-0043] Umber, M., Gomez, R.‐M., Gélabale, S., Bonheur, L., Pavis, C. & Teycheney, P.‐Y. (2017) The genome sequence of Dioscorea bacilliform TR virus, a member of the genus *Badnavirus* infecting *Dioscorea* spp., sheds light on the possible function of endogenous *Dioscorea* bacilliform viruses. Archives of Virology, 162, 517–521. 27770216 10.1007/s00705-016-3113-3

