# Supplementary material for: Detection and diversity of viruses infecting African yam (Dioscorea rotundata) in a collection and F1 progenies in Côte d'Ivoire shed light to plant‐to‐plant viral transmission
Source: Plant Pathol. 2021 May 14;70(6):1486–95. doi: 10.1111/ppa.13393 (PMC8360134; doi:10.1111/ppa.13393)
Supplement: Supplementary file 3 — Table S3 [file PPA-70-1486-s001.docx]

TABLE S3: Sequences of the eight primer pairs used for PCR-based detection tests of yam viruses.

| **Name** | **Targeted virus** | **Sequence** | **Reference** |
| --- | --- | --- | --- |
| CMV1-F  CMV1-R^a^ | CMV | 5'-GTA GAC ATC TGT GAC GCG A-3'  5'-GCG CGA AAC AAG CTT CTT ATC-3' | De Blas *et al*., 1994 |
| Seco-1F  Seco-1R^a^ | DMaV | 5’AAC TCC WTC WGG WTT YGC TYT GAC 3’  5’ CCC ACT TYC TYT TGA GAA AAT CAA 3’ | Umber *et al*., 2020 |
| DiosClos-F  DiosClos-R^a^ | YaV1 | 5’CTC TTT AGG TTT CCC ATT TAT CA 3’  5’ TGG TTC TAC ATT ACT AGA CTA C 3’ | Marais *et al*., 2020 |
| YMV1  YMV2^a^ | YMV | 5’TGC GGA ACT CRA AAG AAC 3’  5’ TGC CAT CAA ATC CAA ACA 3’ | Bousalem *et al*., 2000 |
| YMMV CP 2F  YMMV UTR 1R^a^ | YMMV | 5’ GGC ACA CAT GCA AAT GAA AGC 3’  5’ CAC CAG TAG AGT GAA CAT AG 3’ | Mumford & Seal, 1997 |
| Badna-FP  Badna-RP^b^ | badnaviruses | 5’ ATG CCI TTY GGI ITI AAR AAY GCI CC 3’  5’ CCA YTT RCA IAC ISC ICC CCA ICC 3’ | Yang *et al*., 2003 |
| YamMac4F  YamMac5R^c^ | Yam  macluraviruses | 5’ CHG CAG CWA TYG GKM GTG 3’  5’ GGG TTG CTG AGC RTY GGA 3’ | Umber *et al*., 2020 |
| Potex 2RC  Potex 5^b^ | Yam  potexviruses | 5’ AGC ATR GCN SCR TCY TG 3’  5’ CAY CAR CA R GCM AAR GAY GA 3’ | Van der Vlugt *et al*., 2002 |
| YamX-3F  YamX-5R^c^ |  | 5’ CIC ART GGG TIA AGA AAR WKG A 3’  5’ GRT CRA AIG CTG TRA ART CAT TIG C 3’ | Umber *et al*., 2020 |

^a^ Specific detection primers; ^b^ Generic detection primers; ^c^ Generic detection primers for yam viruses.

De Blas, C., Borja, M.J., Saiz, M and Romero, J. (1994) Broad spectrum detection of cucumber mosaic virus (CMV) using the polymerase chain reaction. *Journal of Phytopathology*, 141, 323-329. https://doi.org/10.1111/j.1439-0434.1994.tb01476.x

Umber, M., Filloux, D., Gélabale, S., Gomez, R.M., Marais, A., Gallet, S. et al. (2020) Molecular viral diagnosis and sanitation of yam genetic resources: implications for safe yam germplasm exchange. *Viruses*, 12, 1101. <https://doi.org/10.3390/v12101101>

Marais, A., Umber, M., Filloux, D., Gomez, R.M., Faure, C., Pavis, C. et al. (2020) Yam asymptomatic virus 1, a novel virus infecting yams (*Dioscorea* spp.) with significant prevalence in a germplasm collection. *Archives of Virology*, <https://doi.org/10.1007/s00705-020-04787-0>

Bousalem, M., Dallot, S., and Guyader, S. (2000) The use of phylogenetic data to develop molecular tools for the detection and genotyping of *Yam mosaic virus*. Potential application in molecular epidemiology. *Journal of Virological Methods*, 90, 25-36. <https://doi.org/10.1016/S0166-0934(00)00198-1>

Mumford, R.A and Seal, S.E. (1997) Rapid single-tube immunocapture RT-PCR for the detection of two yam potyviruses. *Journal of Virological Methods*, 69, 73-79. https://doi.org/10.1016/S0166-0934(97)00141-9

Yang, I.C., Hafner, G.J., Revill, P.A., Dale, J.L and Harding, R.M. (2003) Sequence diversity of South Pacific isolates of Taro bacilliform virus and the development of a PCR-based diagnostic test. *Archives of Virology*, 148, 1957-1968. <https://doi.org/10.1007/s00705-003-0163-0>

Van der Vlugt, R.A.A. and Berendsen, M. (2002) Development of a general potexvirus detection method. *European Journal of Plant Pathology*, 108, 367-371. https://doi.org/10.1023/A:1015644409484
